# Complete mitochondrial genome and phylogenic analysis of *Rhinogobius cliffordpopei* (Perciformes, Gobiidae)

**DOI:** 10.1080/23802359.2019.1637287

**Published:** 2019-07-12

**Authors:** Dan Wang, Chaoxu Dai, Qiang Li, Yahong Li, Zhizhi Liu

**Affiliations:** a Key Laboratory of Exploration and Utilization of Aquatic Genetic Resources, Ministry of Education, Shanghai Ocean University, Shanghai, China;; b National Demonstration Center for Experimental Fisheries Science Education, Shanghai Ocean University, Shanghai, China;; c Aquatic Animal Genetics and Breeding Shanghai Cooperative Innovation Center, Shanghai Ocean University, Shanghai, China

**Keywords:** *Rhinogobius cliffordpopei*, mitochondrial genome, phylogenetic analysis

## Abstract

The *Rhinogobius* (Perciformes, Gobiidae), is a species-rich freshwater gobiid genus. In this article, the complete mitochondrial genome (mtDNA) of the *Rhinogobius cliffordpopei* was first determined. The mtDNA of *R. cliffordpopei* consisted of 13 protein-coding genes, 22 tRNA genes, 2 rRNA genes, and a control region. In the phylogenetic tree, *R. cliffordpopei* firstly joined with *R. brunneus*, and next clustered with *R. giurinus*. Then, they constituted Gobiidae clade with the rest other six species. This relationship consists of taxonomic status. The results would provide basal molecular data for the future research of adaptive evolution in *Rhinogobius*.

The *Rhinogobius* is a species-rich freshwater gobiid genus, extensively distributed in eastern Eurasia and some islands of the western Pacific. It is the largest genus of freshwater gobies in this area, involving more than 85 species and 39 genera distributed in China (Suzuki et al. 2004; Oijen et al. 2011). *Rhinogobius cliffordpopei* is a pint-size benthic fish, distributed in the Liao River, Yangtze River, and Qiantang River. *Rhinogobius cliffordpopei* is a small-sized fish and the body length of sexually matured individuals is normally 30 mm. Because of its high environmental adaptability and being lack of suitable predator *R. cliffordpopei* has bred a large population in many glasses of water now and threatened the growth and survival of native fish. Therefore, the fish has attracted more and more attention of ecologists and evolutionary biologists. It is time for people to take measures to control the fish’s reproduction. However, only a few kinds of research have studied on it (Wu and Zhong 2008; Kondo et al. 2013). So, we determined the mtDNA of *R. cliffordpopei* for the first time and compared with the mtDNA (GenBank: KP 892753) of *Rhinogobius giurinus*, which has highly similar phenotypes with the former. To do so, we wanted to find suitable molecular markers for the two species and provide useful basal information for the adaptive evolution research of Gobiidae in the future.

The sample was collected from the Qiantang River, Zhejiang province, China. The samples were stored in 75% ethanol at 4 °C in Fish Herbarium of Shanghai Ocean University. We used phenol/chloroform to extract the genomic DNA. Firstly, three PCR primers were initially obtained from similarity species, which were used to amplify the complete mitochondrial genome sequence. Secondly, the other six primers were designed according to obtained sequences to supplement residual gaps. DNA Baser V.3.5.4 (http://www.dnabaser.com/news/index.html) was used to assemble the complete sequence. The whole mitochondrial genome was annotated by MitoFish and Mitoannotator (Iwasaki et al. 2013) and then submitted to GenBank. All tRNA genes were recognized by tRNAsan-SE1.21 (Lowe and Eddy 1997), which was also used to characterize the anti-codons of all tRNAs. We used MEGA 7.0 to figure out the base composition. To further study the phylogenetic position of *R. cliffordpopei*, 16 similar species’ mtDNA were collected from GenBank. Firstly, the mitochondrial sequences were aligned using clustal W and the sequence adjusted manually. Secondly, Jmodel Test2 (Darriba et al. 2012) was employed to estimate the best DNA model, according to a smaller AIC date. Then, we used maximum-likelihood (ML) to reconstruct phylogenetic trees and the number of bootstrap replicates was 1000.

The complete mitochondrial genome of *R. cliffordpopei* was 16,525 bp in length (GenBank number: KT 357638). It was composed of 13 typical protein-coding genes, 22 tRNA genes, 2 rRNA genes, and a control region. Besides *ND6* and eight tRNA genes, other genes were encoded on the heavy strand. *COI* gene initiated with GTG, whereas the other protein-coding genes initiated with ATG. Two stop codons (TAA, TAG) and three incomplete stop codons (TAA, TA, T) were found in the protein-coding genes. The length of 22 tRNA genes ranged from 62–75 bp. The genes of 12s rRNAs and 16s rRNAs were separated by the tRNA^phe^, and tRNA^leu^. The control region was located between the tRNA^pro^ and tRNA^phe^. The content A + T (50.8%) was higher than the content G + C (49.2%), which was similar to other vertebrates (Li and Liu 2016). The upper mtDNA organization and structure of *R. cliffordpopei* is very similar to *R. giurinus’*. The two mtDNA had 2363 variation sites, accounting for 15%. Especially, there was a 10–22% variation in the protein-coding genes. The analysis showed that *ND4* and *ND5* genes had the most mutation loci and they would be useful molecular markers for identification of the two morphological similarity fish.

Altogether, 18 typical Gobies were used to construct a phylogenetic tree. In Figure 1, *R. cliffordpopei* firstly joined with *R. brunneus*, and next clustered with *R. giurinus.* Then, the nine species constituted Gobiidae clade. Finally, Gobiidae, Eleotridae, Rhyacichthyidae, and Odontobutidae formed sister groups. This relationship consists of taxonomic status (Wang et al. 2001; Thacker 2009; Agorreta and Rüber 2012; Tornabene et al. 2013).

**Figure 1.**
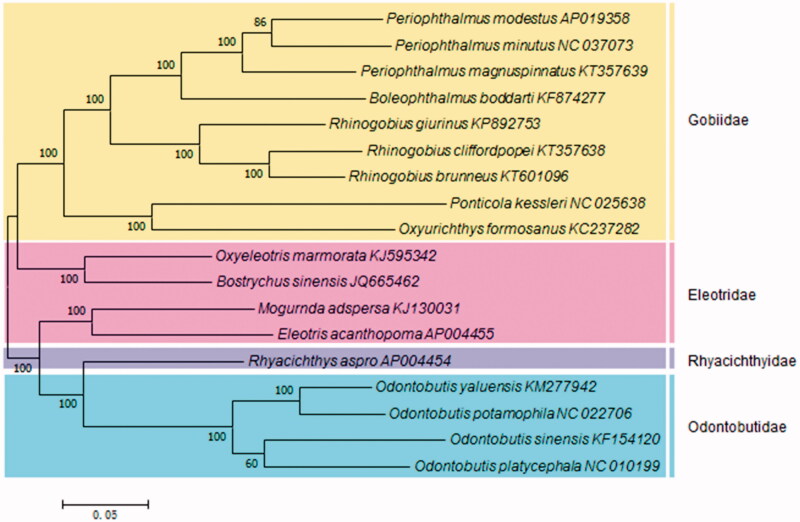
The phylogenetic relationship for fish of the Gobiidei.
